# Supplementary material for: TiO2 NPs-immobilized silica granules: New insight for nano catalyst fixation for hydrogen generation and sustained wastewater treatment
Source: PLoS One. 2023 Jun 21;18(6):e0287424. doi: 10.1371/journal.pone.0287424 (PMC10284393; doi:10.1371/journal.pone.0287424)
Supplement: Attachment — Submitted filename: Manuscript ID_PONE-D-23-10591.docx [file pone.0287424.s001.docx]

Manuscript ID: PONE-D-23-10591

Title: TiO_2_ NPs-immobilized Silica Granules: New Insight for Nano Catalyst Fixation for

Hydrogen Generation and Sustained Wastewater Treatment

In this manuscript, the authors desribe a novel method for immobilizing R25 nanoparticles on silica granules using hydrothermal treatment and calcination was introduced for heterogeneous catalytic processes. The resulting composite showed good performance in removing methylene blue dye and improved photocatalytic activity for hydrogen generation from sewage wastewaters under direct sunlight. The topic of the paper is interesting but the manuscript contains information and some propositions that are not well-justified. Furthermore, additional experiments are required to evaluate the catalytic performance of the as-prepared material. Therefore, the paper cannot be accepted for publication in its present form and major revision is needed. Some suggestions were listed as follows:

1. Abstract and introduction sections should be concisely rewritten to emphasize and highlight the results and urgency of this work.

2. In this work, the authors used a hydrothermal process for fabricating materials, so the hydrothermal method should be introduced, and some previous studies (such as <https://doi.org/10.1016/j.ijhydene.2018.09.174>, <https://doi.org/10.1166/jnn.2018.15719>) should be mentioned as examples for advantages of the hydrothermal route.

3. The authors investigated the effect of temperature (140, 150, 170, and 185 ^o^C) on the formations of the catalysts by the hydrothermal routes, so the authors should be showed the change of behaviors of catalysts according to the temperatures. Why the authors did not investigate the effect of reaction time, which is a vital factor in fabricating the materials?

4. The particle size of TiO_2_ before and after also should be confirmed by its effect on the catalytic performance. In addition, can the author control the TiO_2_ structure?

5. In photocatalysis applications, the band gap, and surface area are important parameters to improve the photocatalytic efficiency, so the authors should be determined such values of the as-made catalysts.

6. The photocatalytic mechanism of the as-obtained photocatalysts should be mentioned. Some references such as <https://doi.org/10.1021/acs.jpcc.0c03590>; <https://doi.org/10.1002/ceat.202200388> should be mentioned when discussing the photocatalytic mechanism.

7. Did the authors investigate the reusability of the catalyst for the photocatalytic degradation of dyes?

8. Some typos of English in the whole manuscript should be re-checked and improved.
